# Driving up the Electrocatalytic Performance for Carbon Dioxide Conversion through Interface Tuning in Graphene Oxide–Bismuth Oxide Nanocomposites

**DOI:** 10.1021/acsaem.2c02013

**Published:** 2022-10-20

**Authors:** Michele Melchionna, Miriam Moro, Simone Adorinni, Lucia Nasi, Sara Colussi, Lorenzo Poggini, Silvia Marchesan, Giovanni Valenti, Francesco Paolucci, Maurizio Prato, Paolo Fornasiero

**Affiliations:** † Department of Chemical and Pharmaceutical Sciences, University of Trieste and Consortium INSTM, Via L. Giorgieri 1, 34127Trieste, Italy; ‡ Department of Chemistry “Giacomo Ciamician”, University of Bologna and Consortium INSTM, via Selmi 2, 40126Bologna, Italy; § CNR-IMEM Institute, Parco area delle Scienze 37/A, 43124Parma, Italy; ∥ Department Politecnico, University of Udine, Unità di Ricerca INSTM Udine, Via del Cotonificio 108, 33100Udine, Italy; ⊥ Institute of Chemistry of Organometallic Compounds, National Research Council of Italy (ICCOM-CNR), Via Madonna del Piano 10, 50019Sesto Fiorentino, Florence, Italy; # Carbon Nanobiotechnology Laboratory, CIC biomaGUNE, Paseo de Miramón 182, 20009Donostia-San Sebastian, Spain; ∇ Ikerbasque, Basque Foundation for Science, 48013Bilbao, Spain; ○ ICCOM-CNR, University of Trieste, Via L. Giorgieri 1, 34127Trieste, Italy

**Keywords:** graphene oxide, bismuth oxide, carbon dioxide reduction, electrocatalysis, interfaces

## Abstract

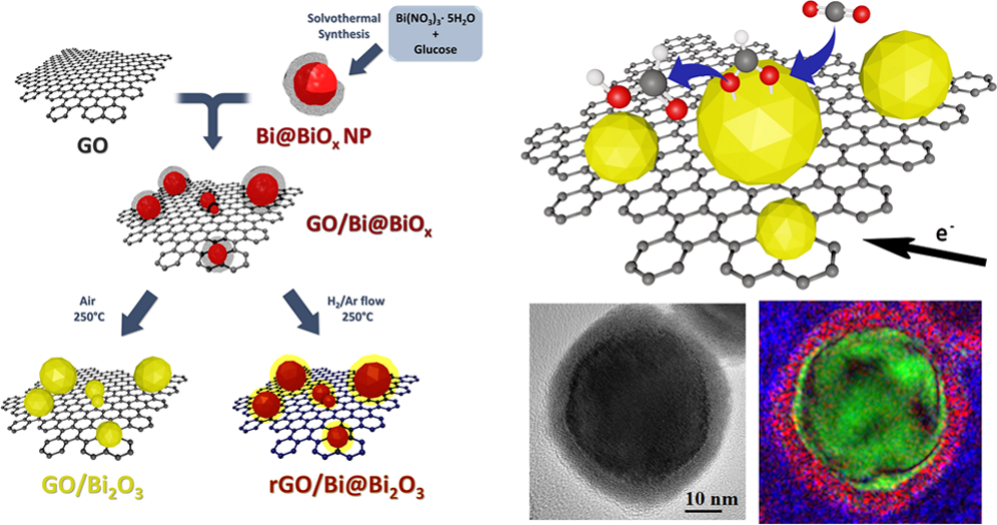

The integration of graphene oxide (GO) into nanostructured Bi_2_O_3_ electrocatalysts for CO_2_ reduction (CO_2_RR) brings up remarkable improvements in terms of performance toward formic acid (HCOOH) production. The GO scaffold is able to facilitate electron transfers toward the active Bi_2_O_3_ phase, amending for the high metal oxide (MO) intrinsic electric resistance, resulting in activation of the CO_2_ with smaller overpotential. Herein, the structure of the GO-MO nanocomposite is tailored according to two synthetic protocols, giving rise to two different nanostructures, one featuring reduced GO (rGO) supporting Bi@Bi_2_O_3_ core–shell nanoparticles (NP) and the other GO supporting fully oxidized Bi_2_O_3_ NP. The two structures differentiate in terms of electrocatalytic behavior, suggesting the importance of constructing a suitable interface between the nanocarbon and the MO, as well as between MO and metal.

## Introduction

A switch to sustainable energy and chemical production is in urgent demand, triggering a vast amount of research toward the implementation of new schemes with low environmental impacts. In this context, the electrochemical reduction of carbon dioxide (CO_2_RR) into value-added products is a highly desirable strategy to create a closed carbon cycle for fuels and chemicals.^1,2^ The CO_2_RR can proceed with a variety of possible products, including the thermodynamically more favored H_2_ evolution (HER) when the reaction is carried out in aqueous electrolytes.^3−5^ This aspect is the main driving force for the continuous development of new selective and active CO_2_RR electrocatalysts able to operate in aqueous environment. Bismuth-based heterogeneous catalysts have flourished in recent years as attractive CO_2_RR electrocatalysts, leading mainly to the formation of formic acid (HCOOH), a very useful feedstock for the chemical and fuel cell industry.^6,7^ Most studies focused on metallic Bi(0), whose typical selectivity toward HCOOH^8−12^ arises from the favorable adsorption of the *OCHO intermediate onto the Bi surface in comparison with *COOH and *H.^10,12^ Nevertheless, downsides are identified with the usually large required overpotentials and modest current densities,^9,13−15^ as well as short operation time stability.^16,17^ These shortcomings inspired the search for engineered Bi-based catalyst structures, in order to achieve CO_2_RR at small overpotential with competitive intrinsic activity and selectivity. During our exploration of electrocatalytic CO_2_RR, we have provided evidence on how the integration of carbon nanostructures (CNS) into carbon/metal oxide (MO) nanocomposite can harness a cascade of events that enables the catalytically active site to be active at earlier onset potentials with increased selectivity toward HCOOH.^18,19^ This synergistic effect arises from the formation of suitable carbon-inorganic interfaces, whereby the CNS is able to (1) improve charge transfers at the interface with the metal/metal oxide, (2) add up to the catalyst’s stability, and (3) facilitate reactant diffusion to the active site, typically by increasing the available surface area and favoring the adsorption capacity of CO_2_.^20,21^ Herein, we chose to explore graphene oxide (GO) as the nanocarbon phase because of its large two-dimensional (2D) morphology, which allows optimum deposition of the MO nanoparticles with no size limitations, and also to focus on the electronic effects, ruling out contributions from the high surface area that could arise with higher surface area CNS such as carbon nanohorns (CNH) or carbon nanotubes (CNT). The use of conductive carbon matrices has been proven to be a proficient strategy to improve current densities of Bi_2_O_3_ electrocatalysts, although selectivity toward HCOOH remained limited to large applied overpotentials,^22^ so that additional complexity must be considered in Bi_2_O_3_-based hybrid catalyst design. Recent reported studies illustrated the role of oxidation of Bi to Bi_2_O_3_ and how this causes a more pronounced CO_2_ adsorption and a quicker first electron transfer step to form the initial CO_2_^–^ radical intermediate, leading to enhanced catalytic performances.^23^ Hence, we explore the synergistic combination of GO and Bi_2_O_3_ toward improved CO_2_RR, with activity boosted by the competent electron mediating role of the CNS, which allows faster kinetics.

The GO/Bi_2_O_3_ nanocomposite, assembled by exploiting the GO oxygenated functional groups for better binding of the MO phase, exhibits different CO_2_RR catalytic behavior depending on the specific structural evolution during the synthetic protocol. The observed electrocatalytic behavior in relation to the nanocomposite structure allows one to uncover many of the subtle shades that strongly affect the complex dynamics of CO_2_RR driven by carbon–MO interfaces. In particular, we analyzed the correlation of the CO_2_RR performance when the inorganic nanoparticles (NP) evolve from a Bi@BiO_*x*_ core–shell configuration featuring an amorphous BiO_*x*_ shell to (i) a fully oxidized and partially crystalline β-Bi_2_O_3_ NPs interfaced with GO or (ii) a truly Bi@β-Bi_2_O_3_ core–shell NP interfaced with reduced GO (rGO) obtained via a reductive thermal treatment. The obtained CO_2_RR correlation indicates significant differences, and will help to establish a platform for the rational design of future and advanced multiphase CO_2_RR electrocatalysts integrating CNS.

## Experimental Section

### Synthesis of GO–Bismuth Oxide Nanocomposites

The GO was prepared following a procedure we previously adopted.^24^ First, 200 mg of pristine graphene was added into a 100 mL round-bottomed flask containing K_2_S_2_O_8_ (200 mg, 0.37 mmol), P_2_O_5_ (100 mg, 0.35 mmol), and H_2_SO_4_ (10 mL). The reaction was sonicated for 30 min and stirred at 80 °C for 4 h. The crude was cooled down, diluted with deionized water (50 mL), filtered through a Millipore membrane (JHWP, 0.45 μm), and washed with deionized water until neutralization of the washings. The black powder collected from the Millipore membrane was transferred into a round-bottom flask and dispersed in 20 mL of H_2_SO_4_ at 0 °C. Then, 100 mg of KMnO_4_ (0.63 mmol) were added into the dispersion under stirring. The final was stirred at 35 °C for 2 h. After that, deionized water (20 mL) and H_2_O_2_ 30% (2.4 mL) were added, and the reaction was stirred for 15 min. The crude was filtered through a Millipore membrane (JHWP, 0.45 μm) and washed with HCl 1 M (100 mL) and deionized water until neutralization of the washings occurred. Finally, the black powder was washed one time with MeOH and dried with Et_2_O, affording 210 mg of GO.

#### Bi@BiO_*x*_ NP

The pristine core–shell nanoparticles were prepared via a solvothermal method adapting a reported procedure.^25^ Bi(NO_3_)_3_·5H_2_O and glucose in molar ratio (1:1.5) were dissolved in ethylene glycol so to have a concentration of 0.067 and 0.1 M, respectively. The solution was stirred for 30 min and then transferred to a Teflon-lined stainless autoclave, and it was heated to 120 °C for 12 h. The black solid was recovered by centrifugation and washed successively with H_2_O and ethanol.

#### Bi@Bi_2_O_3_ NP

The as prepared Bi@BiO_*x*_ NP samples were subjected to calcination at 250 °C under static air atmosphere, with a ramp of 3 °C min^–1^.

#### GO/Bi@BiO_*x*_

GO and Bi@BiO_*x*_ NP were prepared in different weight ratios (3:1, 4:1, 5:1). The procedure involved the dispersion under sonication of the two solids in ethanol so to have approximately a 2:1 mg mL^–1^ dispersion of GO. After the sonication, the mixture was stirred at room temperature for 24 h. The solid was recovered by centrifugation.

#### GO/Bi_2_O_3_

The as prepared GO/Bi@BiO_*x*_ samples were subjected to calcination at 250 °C under static air atmosphere, with a ramp of 3 °C min^–1^.

#### rGO/Bi@Bi_2_O_3_

The as prepared GO/Bi@BiO_*x*_ samples were subjected to thermal treatment at 250 °C in a tubular furnace under a flowing stream of a mixture of H_2_/Ar (230 mL min^–1^, with 30 mL of H_2_ and 200 mL of Ar).

### Materials Physical Characterization

Raman spectra were recorded with an Invia Renishaw microspectrometer equipped with He–Ne laser at 532 nm (1% power). At least 10 spectra per sample in different spots were collected to check their homogeneity. X-ray diffraction (XRD) was performed on a Philips X’Pert diffractometer using a monochromatized Cu Kα (λ= 0.154 nm) X-ray source in the range 10° < 2θ < 100. X-ray photoelectron spectroscopic (XPS) analyses were performed in an UHV chamber with a base pressure lower than 10^–9^/10^–10^mbar. The chamber was equipped with nonmonochromatized Al radiation (*hυ* = 1486.6 eV) and a hemispherical electron/ion energy analyzer (VSW mounting a 16-channel detector). The operating power of the X-ray source was 1440 W (12 kV and 12 mA) and photoelectrons were collected normal to the sample surface, maintaining the analyzer angle between analyzer axis and X-ray source fixed at 54.5°. All the samples were adsorbed on aluminum foil, and XPS spectra were acquired in a fixed analyzer transmission mode with a pass energy of 44.0 eV. The spectra were analyzed by using the CasaXPS software. Shirley functions have been used to subtract the background. The deconvolution of the XPS spectra has been carried out employing a Lorentzian asymmetric, and the binding energies (B.E.) were calibrated upon fixing the Al 2p component of Al(0) at 71.8 eV. Transmission electron microscopy (TEM) was performed using a JEOL 2200FS microscope working at 200 kV equipped with an energy dispersive X-ray spectrometer (EDX), a high-angle annular dark-field (HAADF) detector, and an in-column energy Omega filter. EDX maps were obtained in scanning TEM (STEM) mode.

### Electrochemical Characterization

The electrochemical characterization was performed with a SP-300 bipotentiostat (*Biologic Instruments*) workstation, using a three-electrode system composed of a saturated calomel electrode (SCE) as the reference electrode, a Pt wire as the counter electrode, and a catalyst-modified glassy carbon electrode (GCE, 3 mm in diameter, geometric surface area 0.071 cm^2^) as the working electrode. The electrochemical characterization was realized in Ar-saturated KOH 0.1 M electrolyte.

To study the electrochemical proprieties of Bi_2_O_3_ composites materials, a thin film was deposited on glassy carbon electrode through drop-casting. For this work, a constant loading of electrocatalyst was used, equal to 505 μg cm^–2^. To deposit the materials, it was necessary to disperse them in suitable solvent: the GO/Bi_2_O_3_ were dispersed in ink of two parts of Milli-Q water and one part of 2-propanol and 0.5% Nafion with a concentration of 1.6 mg mL^–1^. For all electrochemical measurements, the potentials were reported versus RHE and corrected for ohmic drop. The quantity of electrocatalyst deposited and the electrochemical activity comparison were made by normalizing by the Bi oxide amount of each electrocatalyst, which was measured by thermogravimetric analysis (TGA) (Figure S8).

### CO_2_RR Characterization

A SP-150 potentiostat (*Biologic Instruments*) workstation and a custom-made electrochemical cell^1^ with three-electrode configuration were used to determine the properties of Bi_2_O_3_ composites for the CO_2_ reduction reaction (CO_2_RR). The innovation of this custom-made cell is the WE placed face-up in the bottom of cell, and the CE (mesh Pt) separated from the electrolyte by porous frit. With this configuration, the gas products can go directly toward the gas chromatograph (GC) for detection, and due to the separate compartment, the liquid products of CO_2_RR cannot react with CE. An Ag/AgCl was used as a reference electrode (LowProfile 3.5 mm OD of *PINE research*). The peculiarity of this electrode is the use of a gel instead of a KCl solution. The gel and the ceramic porous frit guarantee a low mobility of the chloride ions, preventing them from escaping from the electrode with consequent poisoning of the catalyst.

The electrolyses were performed in a near-neutral bicarbonate buffer, KHCO_3_ 0.5 M. This electrolyte was pre-electrolyzed before use to guarantee high purity, as it is known that even small amounts of metal impurities can lead to surface interference with electrochemical reactions. Pre-electrolysis was carried out in a cell with a two-electrodes configuration, with Pt wire as a counter electrode and a Pt mesh as a working electrode; electrolysis of the electrolyte was performed for at least 24 h at a current of 0.1 mA while stirring the solution, which was Ar-saturated.^2^

As for electrochemical characterization, the same inks and loading of materials were used for the CO_2_RR study, but in this case, a bigger electrode area was used (GCE, geometric surface area 1 cm^2^). The electrode area is important because the larger the electrode area, the higher the concentration of the reaction products. In this way the detection limit of the instrument (GC) is exceeded, guaranteeing the reliability and reproducibility of the measurements.

The CO_2_RR activity was evaluated by chronoamperometry (CA) of 1 h and 51 min in CO_2_-saturated electrolytes. The gaseous products were analyzed during measurements by online gas chromatography (GC) directly connecting the headspace of the electrochemical cell to the sample loop of a GC, while formic acid was detected by analysis of the liquid phase by ionic chromatography (IC) at the end of electrolysis. The gas phase quantification was carried out during the electrolysis with sampling every 15 min. The Faradaic efficiency (FE) for the gas products of CO_2_RR was quantified following the procedure previously described by Baltrusaitis et al.^3^ (eq 1)
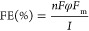
1where *n* is the number of electrons needed for CO_2_RR; *F* is the Faraday constant; φ is the volume fraction of the gas; *I* is the current, and *F*_m_ is the molar CO_2_ gas flow rate.

While the analyses of the liquid products were performed by means of a Metrohm model 850 Professional IC Ion Chromatograph equipped with a Metrosep A Supp 4-250/4.0 anion column and a conductivity detector. The eluent used was 0.5 mM H2SO4, with 15% acetone. The Faradaic efficiency (FE) for the formic acid products was quantified in the following way (eq 2):
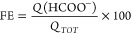
2

## Results and Discussion

### Synthesis and Characterization of GO–Bismuth Oxide Nanocomposites

**Figure 1:**
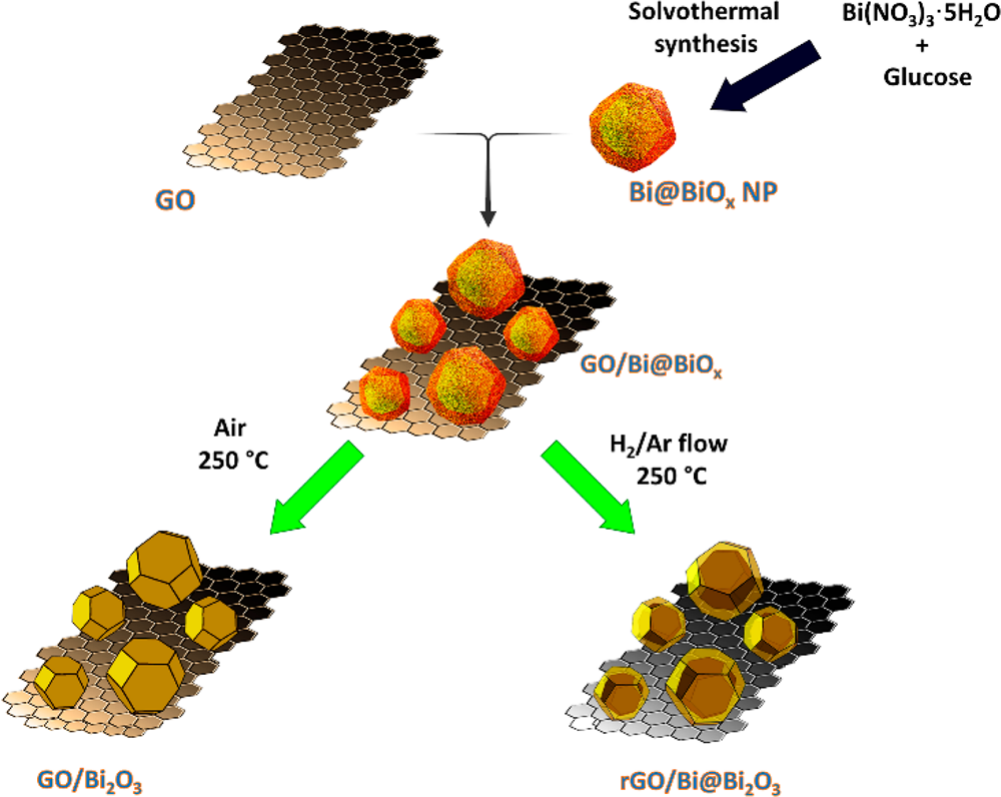
Graphical sketch of the synthesis to obtain the different nanocomposites.

The Bi@BiO_*x*_ NP samples were synthesized via a facile solvothermal method in the presence of glucose as the additional reducing and stabilizing agent.^25^ The as obtained Bi@BiO_*x*_ NP samples were then deposited onto GO in an ethanol dispersion, whereby the presence of BiO_*x*_-adsorbed glucose favors the coupling with the GO surface. The formation of the nanocomposite (GO/Bi@BiO_*x*_) is confirmed by transmission electron microscopy (TEM) investigation, which shows that the Bi@BiO_*x*_ NP samples are well dispersed onto the GO (Figure S1). After separation, the solid was divided into two fractions, which were independently subjected to two different thermal treatments to cause BiO_*x*_ crystallization and removal of organic groups. One treatment consisted in a calcination under static in air at 250 °C (GO/Bi_2_O_3_), while in the other, the GO/Bi@BiO_*x*_ fresh sample, the thermal treatment was performed under a dynamic atmosphere of H_2_/Ar (rGO/Bi@Bi_2_O_3_). Figure 1 shows a sketch of the synthesis to the two final nanocomposites.

**Figure 2:**
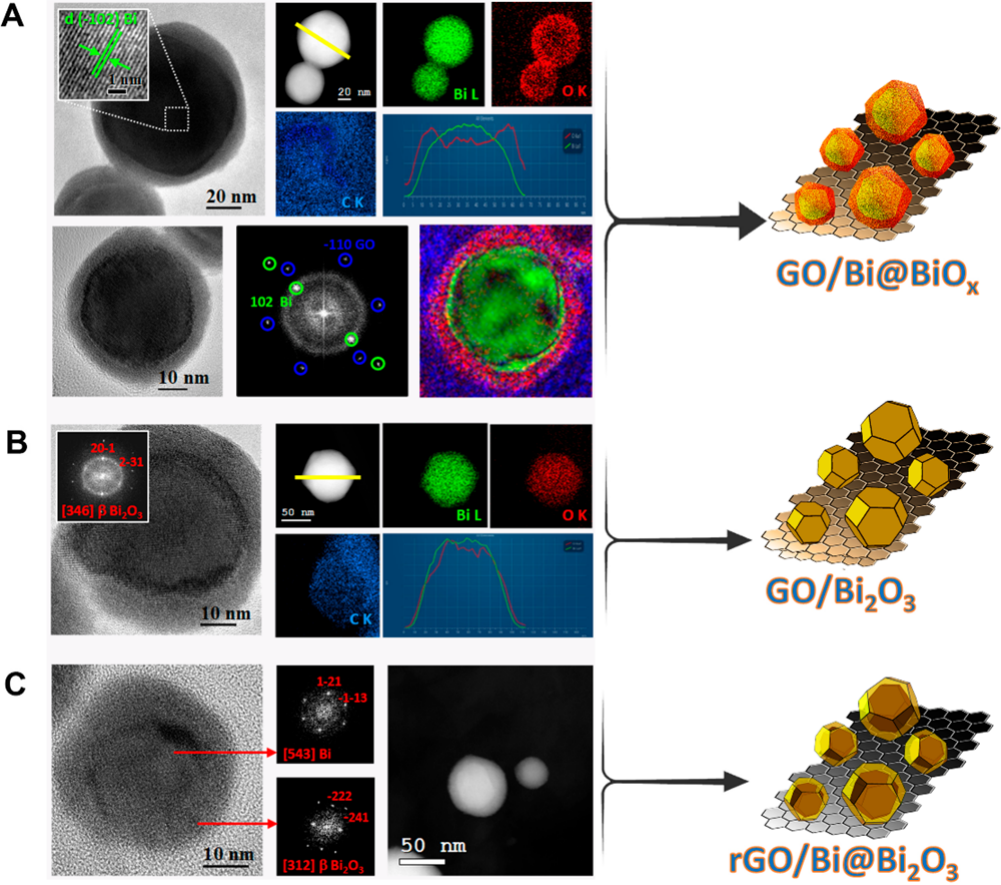
Electron microscopy study of the nanocomposites with graphical sketch of the three nanostructures. (A) Top panels from left to right: representative HRTEM image of GO/Bi@BiO_*x*_ with the inset showing the *d*-spacing (−102) of Bi, HAADF-STEM image with the corresponding C, Bi, and O EDX maps. The EDX line-scan profile across the yellow line evidences the core–shell structure of Bi@Bi_2_O_3_. Bottom panels from left to right: HRTEM of GO/Bi@BiO_*x*_ with corresponding FFT showing the reflections from the (−102) planes of Bi and the [001] zone axis of GO, and the diffuse ring of the amorphous shell, and a color map displaying the inverse FFT generated by selecting the reflections relative to the Bi core (green), the oxide shell (red), and the GO layer (blue). (B) Large panel: HRTEM of GO/Bi_2_O_3_. Inset: FFT of the core showing the [346] zone axis of β-Bi_2_O_3_. Small panels clockwise: HAADF-STEM image of GO/Bi_2_O_3_, with the corresponding C, Bi, and O EDX maps, and EDX line-scan profile across the yellow line. (C) Main panel: HRTEM of rGO/Bi@Bi_2_O_3_ showing the crystalline core/shell structure, Small panels: FFTs of the core and the shell showing the Bi and β-Bi_2_O_3_ phases respectively. Last panel: representative HAADF-STEM image.

**Figure 3:**
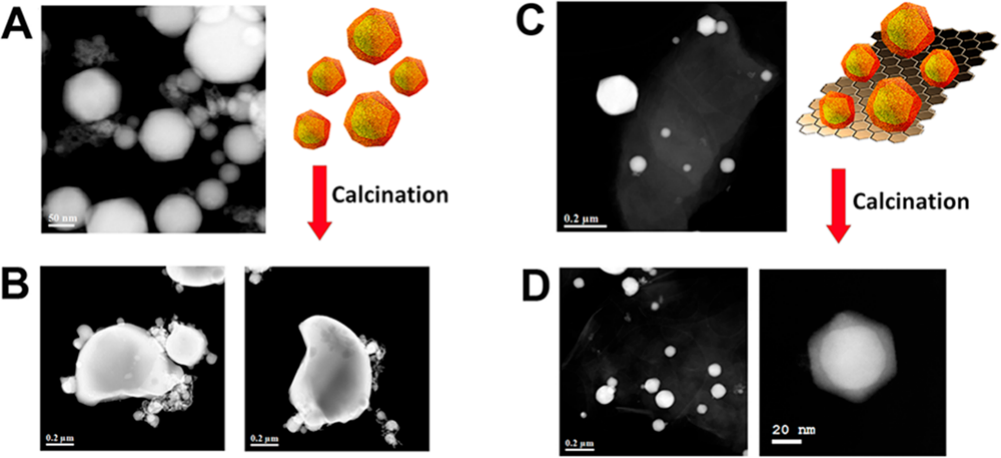
HAADF-STEM images: (A) free-standing Bi@BiO_*x*_ NP, (B) free-standing Bi@BiO_*x*_ after calcination at 250 C (sample Bi_2_O_3_ NP), (C) as prepared GO/Bi@BiO_*x*_, and (D) GO/Bi_2_O_3_.

The evolution of the nanocomposite structures was inspected by high resolution TEM (HRTEM), revealing the core–shell configuration of the inorganic component prior and after combination with GO. In particular, in both Bi@BiO_*x*_ and GO/Bi@BiO_*x*_, the inorganic core consists of rhombohedral metallic Bi, identified by the interplanar *d*-spacing of 0.32 nm, typical of the Bi(012) plane (Figure 2a),^26^ while the shell is made of amorphous BiO_*x*_ (Figure 2 and Figure S2). Interestingly, both the core and shell present a hexagonal geometry (Figure 2a, Figure 3d, and Figure S2). Energy dispersive X-ray analysis (EDX) provides the element mapping of C, O and Bi, and also confirms the core–shell nature of the nanoparticles, deductible from the EDX line profile spectra (Figure 2a, Figure 2b and Figure S3). The calcination treatment causes a full oxidation of the Bi(0) core to bismuth oxide, with partial formation of the metastable β-Bi_2_O_3_, as indicated by the fast Fourier transform (FFT) analysis, as well as by EDX mapping and line profile (Figure 3b). This is also noted by analyzing the HRTEM of a sample made of self-standing Bi_2_O_3_ NP prepared by calcination of the as-prepared Bi@BiO_*x*_ without supporting them on GO (Figure S4). It is noteworthy that, despite the fact that the entire Bi is oxidized to Bi_2_O_3_, the morphology as observed with the TEM retains the memory of the core–shell motif, although in this case both the core and the shell are made of Bi oxide. In contrast, when the treatment is performed in H_2_/Ar, the metallic Bi character of the core is preserved, while the shell becomes oxidized to β-Bi_2_O_3_; thus, a truly Bi@β-Bi_2_O_3_ core–shell structure is established, as also confirmed by the FFT analysis of the core and the shell (Figure 2c). Annular dark-field scanning transmission electron microscopy (HAADF-STEM) analysis provides neater evidence of the core–shell configuration (Figure 2c). It must be noted that in all samples the Bi oxide nanospheres are also surrounded by smaller and thinner fragments of GO, difficult to see with TEM. Nevertheless, accurate HRTEM could reveal such a structural figure, as shown in Figure S5. Moreover, different EDX color contrast on the GO/Bi_2_O_3_ nanocomposite shows that carbon surrounds the whole Bi_2_O_3_ NPs (Figure S6).

Importantly, the reference sample (Bi_2_O_3_ NP) prepared by calcination of the self-standing Bi@BiO_*x*_ NP results in the formation of several large aggregates of the nanoparticles, confirming the stabilizing role of GO, which prevents coalescence of the NP. Figure 3 shows the comparison between the HAADF-STEM images of the self-standing Bi@BiO_*x*_ NP as prepared and postcalcination (Figure 3, parts a and b, respectively) with the ones stabilized by supporting them on GO, namely GO/Bi@BiO_*x*_ and GO/Bi_2_O_3_ (Figure 3, parts c and d, respectively).

**Figure 4:**
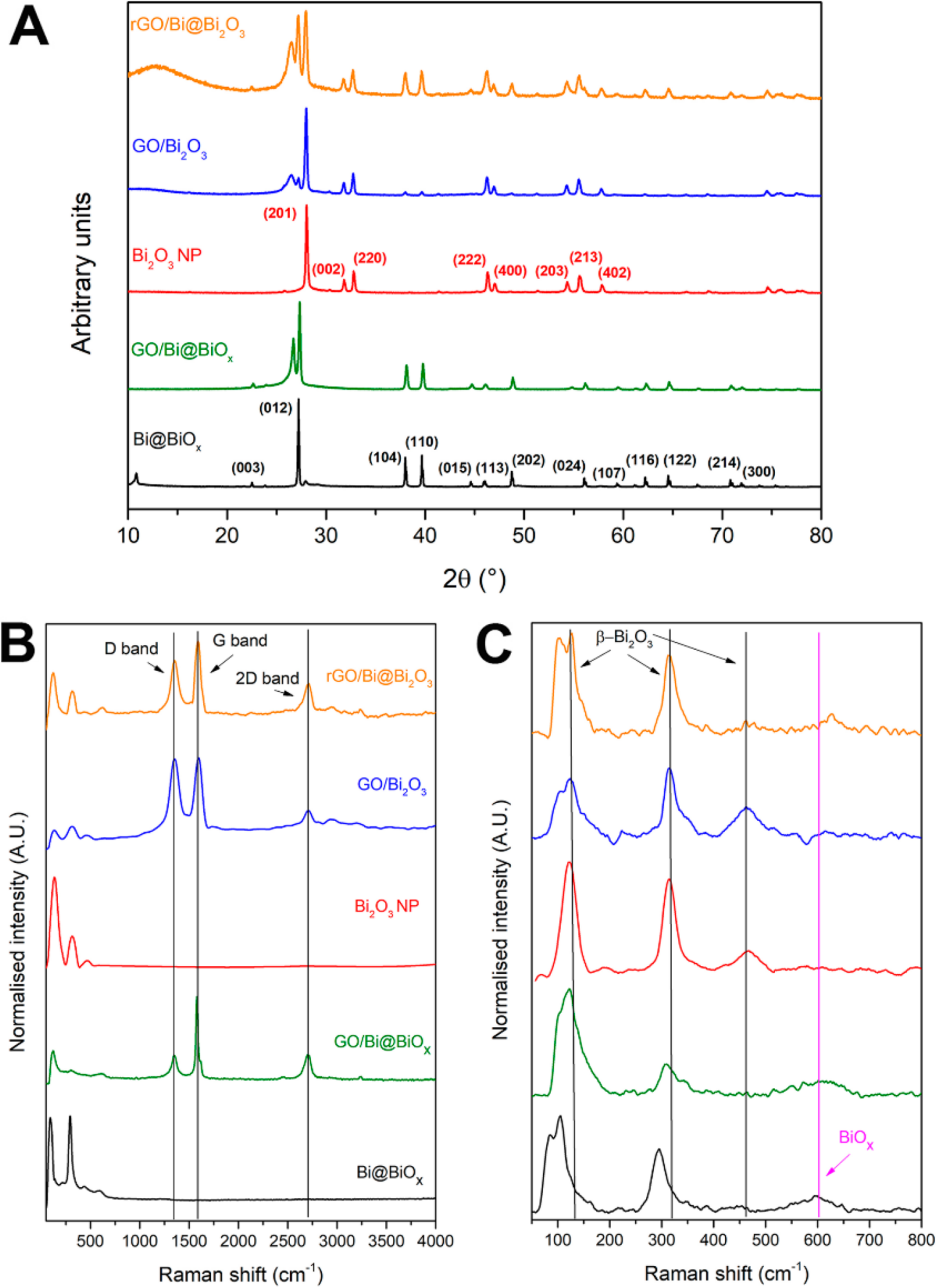
XRD and Raman analysis. (A) Powder XRD patterns of GO, Bi_2_O_3_ NP, Bi@BiO_*x*_, and of all three nanocomposites with crystal reflections identified and indexed to either rombohedral Bi or β-Bi_2_O_3_; (B) full range Raman spectra of GO, Bi_2_O_3_ NP, Bi@BiO_*x*_, and of all three nanocomposites showing also the D, G, and 2D bands relative to GO; (C) Raman in the range 0–800 nm showing the bands relative to β-Bi_2_O_3_ and amorphous BiO_*x*_.

The nanoscale transmission electron microscopy results are in excellent agreement with large-scale X-ray diffraction (XRD) and Raman analysis. In the XRD (Figure 4), the metallic Bi phase is the only one observed for Bi@BiO_*x*_ and GO/Bi@BiO_*x*_, indexed to the rombohedral Bi (JCPDS No. 05-0519), while no relevant Bi_2_O_3_ could be detected, corroborating the amorphous nature of the BiO_*x*_ shell.^25,27^ After the calcination treatment, the Bi(0) core mostly crystallizes forming the β phase of Bi_2_O_3_ (tetragonal) readily indexed to the *P̅*42_1_*c* space group (ICDD crystallographic card number 01-077-5341),^28^ while the reflections of Bi(0) have considerably decreased following such an oxidation. It is worth noting that calcination also causes a broadening of the reflection at ∼27°, typical of graphitic carbon, suggesting that a reduction in the number of stacked layers of the multilayer GO has occurred.^29^ In contrast, the thermal treatment under reducing conditions (rGO/Bi@Bi_2_O_3_) serves to crystallize the BiO_*x*_ shell, while the core preserves its metallic nature, as observed by the coexistence of the reflections assigned to rombohedral Bi and β-Bi_2_O_3_ in the XRD. Additionally, micro-Raman analysis confirms the XRD results. The spectrum of the as-prepared Bi@BiO_*x*_ nanoparticles confirms the presence of the two peaks (respectively at 85 and 105 cm^–1^) which can be assigned to the E_g_ and A_1g_ vibrational modes of Bi–Bi in metallic bismuth, with the frequencies having been slightly blue-shifted with respect to bulk Bi due to the decrease of particle size to the nanoscale.^30^ A less intense peak at 605 cm^–1^ is also observed, attributed to the amorphous BiO_*x*_ phase, perhaps being carbonated following glucose binding,^31^ and confirming that the BiO_*x*_ shell in the noncalcined samples is not crystalline.^32^ The origin of a third well-defined peak at 293 cm^–1^ is not clearly assigned based on literature studies; we hypothesize it is due to some specific Bi–O Raman mode of the amorphous oxide shell still covered by carbonaceous species deriving from glucose. Accordingly, a first remarkable difference after combination with GO is that such a peak is broader and significantly blue-shifted (308 cm^–1^), and the same occurs to the BiO_*x*_ peak that is blue-shifted to 627 cm^–1^. No signal pattern associable to specific Bi_2_O_3_ phases is observed, indicating the amorphous nature of the Bi oxide. We interpret this result as an effect of the electronic interaction between GO and the amorphous BiO_*x*_ shell, in agreement with the X-ray photoelectron spectroscopy (XPS) discussed later on. As anticipated on the basis of XRD, the crystallization of the Bi oxide in GO/Bi_2_O_3_ is verified by the appearance of three new peaks at ca. 123, 315, and 465 cm^–1^ attributed to Bi–O stretching modes in β-Bi_2_O_3_.^31,33−35^ Meanwhile, the signature of GO in both GO/Bi@BiO_*x*_ and GO/Bi_2_O_3_ is clearly visible, with the D and G bands appearing at 1350 and 1570 cm^–1^ respectively, the former related to the A_1g_ breathing mode and the latter, together with another band (2D band) at 2700 cm^–1^, associated with the first- and second-order allowed Raman mode E_2g_.^36^ It is worth noting that both the low D/G bands intensity ratio and the sharpness and intensity of the 2D band indicate that the starting GO has a low level of oxidation and a small disorder, while the calcination treatment induces creation of defects, presumably by insertion of additional oxygen functionalities.^37^ In order to avoid any artifact in the interpretation of the signal shift, we also ran a 80-point micro-Raman mapping of a selected area of the GO/Bi@BiO_*x*_ sample. In such a specific region, the vast majority of the material is the composite (GO/Bi@BiO_*x*_), but there is also a small fraction of stand-alone Bi@BiO_*x*_. The analysis of the spectra confirms that the Raman patterns are different depending on whether the laser is focusing on the nanocomposite or on the isolated Bi@BiO_*x*_ NPs, in particular with the peak at 306 cm^–1^ being shifted when coupled with GO (315 cm^–1^). Moreover, the peak due to amorphous BiO_*x*_ is barely visible in the stand-alone Bi@BiOx and centered at 607 cm^–1^, while is better defined and centered at 627 cm^–1^ in the nanocomposite (Figure S7). Analysis of rGO/Bi@Bi_2_O_3_ indicates that the reduction treatment has a beneficial effects in partially restoring a smaller level of defects, as expected for reduced GO and indicated by the decrease of the D band intensity in relation to the G band.^38^ Moreover, the β phase pattern is also found, together with a peak 103 cm^–1^ characteristic of metallic Bi.

**Figure 5:**
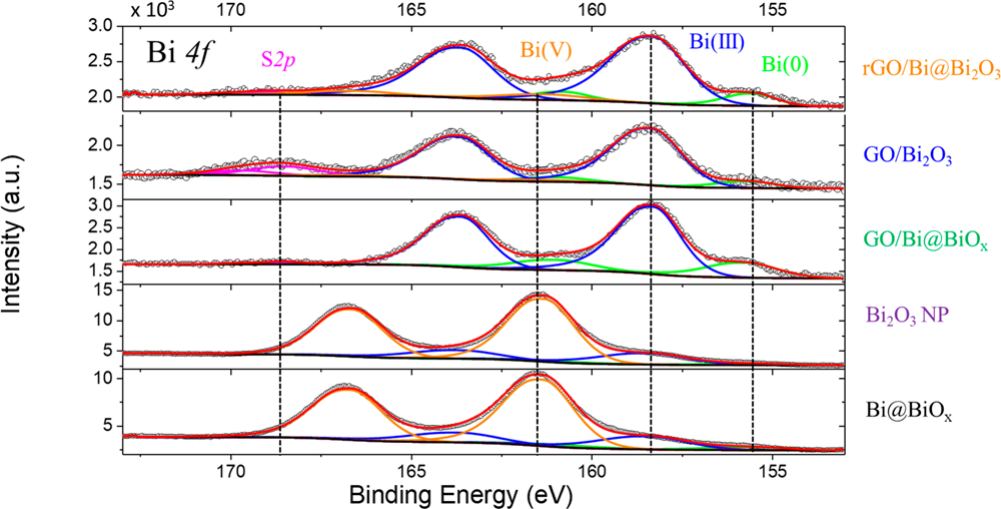
XPS analysis. High-resolution XPS spectra in the Bi 4f region. Colors of the captions are associated with the materials as follows: black (Bi@BiO_*x*_), green (GO/Bi@BiO_*x*_), blue (GO/Bi_2_O_3_), orange (rGO/Bi@Bi_2_O_3_), and purple (Bi_2_O_3_ NP).

X-ray photoelectron spectroscopy (XPS) provides the superficial electronic states of the elements so that it gives a detailed understanding on the interface GO/Bi oxide. XPS confirmed the presence of C and Bi elements in the analyzed samples (Figure 5). The high-resolution XPS of the Bi 4f core level of all the five samples shows the signature doublet of Bi with binding energies (BE) at 159.2 and 164.5 eV associated with the Bi^5+^ 4f_7/2_ and Bi^5+^ 4f_5/2_,^39^ although interesting differences are observed among the samples. In the as-prepared Bi@BiO_*x*_ NP and in the calcined Bi_2_O_3_ NP, the BE of the two main peaks is located at 161.3 eV (orange components of Figure 5) plus its spin–orbit coupling peak at 166.6 eV, in agreement with an oxidation number of the metal of 5+.^40^ The additional presence of two small contributions with BE of 155.5 and 158.4 eV are associated with metallic Bi (Figure 5A, green line)^41,42^ and Bi^3+^.^43−45^ The overall Bi 4f picture is consistent with the small penetration range of XPS, which makes difficult to detect the internal Bi core, while the oxidized shell is readily observed. The fact that the outer shell of the B oxide is overoxidized suggests a tendency of the NPs to react with atmosphere. However, once the Bi@BiO_*x*_ NP are combined with GO (GO/Bi@BiO_*x*_), the doublet is shifted toward lower BE (158.3 and 163.65 eV) confirming, as hypothesized after Raman analysis, that there is an electronic interaction between the GO and Bi@BiO_*x*_ (Figure 5, green line). Calcination of the composite (GO/Bi_2_O_3_) does not alter such a BE shift, implying that even in the crystallized form, the Bi_2_O_3_ is still electronically interacting with GO. The metallic Bi components is still quite visible in the rGO/Bi@Bi_2_O_3_ sample, confirming that the thermal treatment under reductive conditions preserves the metallic Bi, while in the calcined sample, this contribution is very low, in agreement with XRD where the contribution of the Bi(0) is very low. This suggests that GO may contribute to the donation of electron density on the outer Bi atoms constituting the core, favoring their reduction. It is worth stressing that the Bi(0) components change as a function of the different treatment of the samples: the trend of this variation is reported in Table 1. In the Bi 4f region is possible to see a small component at 168.5 eV arising from sulfur impurity. C 1s peaks of GO/Bi@BiO_*x*_ were deconvoluted into four components at 284.0, 285.3, 287.2, and 290.0 eV, plus a shakeup component centered at 293.9 eV, which are ascribed to the sp^2^/sp^3^ carbons, C–OH/C–O–C, C=O bond, and O–C=O respectively, arising from the functional groups of GO (Figure S9).^46−48^ Consistently, in the nanocomposites prior to and after the thermal treatments, the relative intensities of such peaks are changed, as the treatments remove most of the GO oxygenated functional groups. The overall picture indicates that the electronic states of the Bi oxide phase are altered by the presence of GO,^49^ which does not act as a mere inert support for the nanoparticles.

**Table 1:** Semi-Quantitative XPS Relative to the % of Bi(0), Bi(III), and Bi(IV) All over the Total Amount of Bismuth

sample	% Bi(0)	% Bi(III)	% Bi(V)
rGO/Bi@Bi_2_O_3_	12.0	81.0	7.0
GO/Bi_2_O_3_	8.0	86.0	6.0
GO/Bi@BiO_*x*_	20.0	78.0	2.0
Bi@BiOx-F	4.0	19.0	77.0
Bi@BiOx-C	3.0	19.0	78.0

### CO_2_RR Electrocatalytic Performance

**Figure 6:**
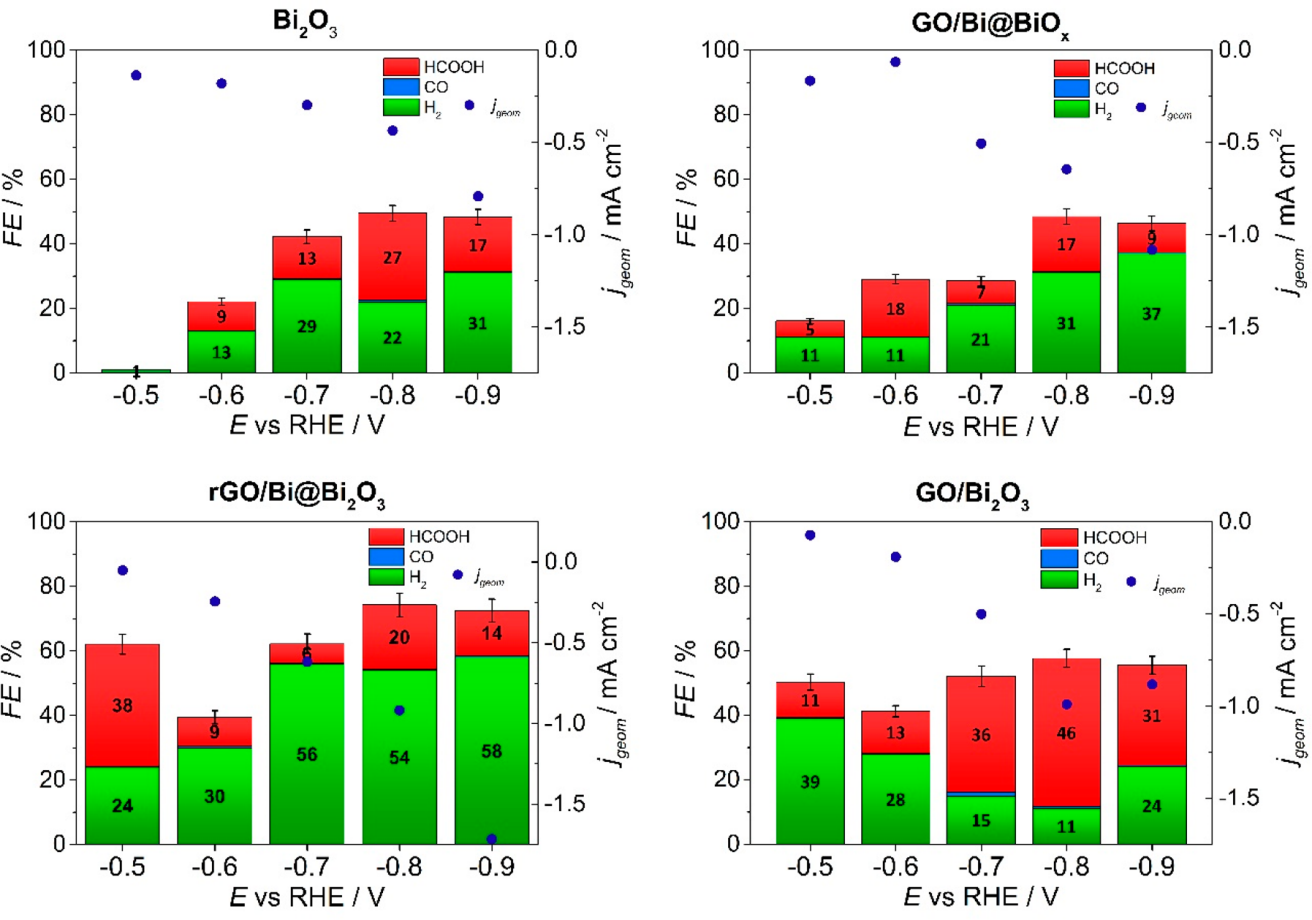
CO_2_RR performance. Faradaic efficiency (FE) of detectable CO_2_RR products in CO_2_-saturated KHCO_3_ 0.5 M (pH 7.5): H_2_ (green), CO (light blue) and HCOOH (red) for (a) Bi_2_O_3_, (b) GO/Bi@BiO_*x*_, (c) rGO/Bi@Bi_2_O_3_, and (d) GO/Bi_2_O_3_. The blue points indicate the mean current density of sample at that potential; the values are shown on the right axis.

The CO_2_RR catalytic activity was evaluated as a function of applied potential, carried out by performing chronoamperometry (CA) experiments in CO_2_-saturated KHCO_3_ for 1 h and 50 min (ranging from −0.4 to −0.9 V vs RHE). Both gas-phase and liquid phase were quantified (the former via gas chromatography, GC, and the latter by ionic chromatography, IC, see the Supporting Information) after the CA in order to determine the product selectivity, reported as the Faraday efficiency (FE). Figure 6 shows, in addition to the FE values, the average values of the involved current density (*j*) during the CA. All the nanocomposites (GO/Bi@BiO_*x*_, GO/Bi_2_O_3_ and rGO/Bi@Bi_2_O_3_) exhibit good catalytic activity for CO_2_RR with HCOOH as the dominant product, with the formation of HCOOH attained at potentials very near the thermodynamic potential.^50^ In all cases, also hydrogen evolution reaction (HER) occurs to different extents depending on the catalyst and the applied potential. The nanocomposites are also compared with the CO_2_RR electrocatalytic performance by GO-free Bi_2_O_3_. Control experiments show that HCOOH production is completely suppressed in the absence of CO_2_, while any possible contribution in the HCOOH production from the nanomaterial is excluded on the basis of the comparison of the CO_2_RR activity with the GO (Figure S10). For all the nanocomposites the presence of GO significantly increases the current density for CO_2_RR and the FE_HCOOH_ (Figure 6) while, importantly, the onset potential is set at earlier values (Figure 9). As we previously reported in the case of HER by oxidized carbon nanotubes/titanium oxide nanocomposites, the carbon–metal oxide turns out less electrocatalytically active in the as prepared nanocomposites. In fact, in the fresh materials, the metal oxide is still in an amorphous state and the CNS presents too many COOH functional groups, leading to a less efficient electronic communication.^51^ This is also observed here in the case of the as prepared GO/Bi@BiO_*x*_ nanocomposite as compared to the two thermally treated catalysts.

In addition, the difference in the hierarchical structures is reflected in significantly different catalytic behavior. The FE_HCOOH_ for rGO/Bi@Bi_2_O_3_ reaches a maximum value of 38% at −0.5 V vs RHE (corresponding to a HCOOH production rate of 3 ppm h^–1^), with a very small overpotential (100 mV), and declines with decreasing the applied potential, while H_2_ is always formed in relatively high FE throughout the explored potential range (part A). In contrast, GO/Bi_2_O_3_ is able to form HCOOH with a higher absolute FE in the more negative range (−0.7 to −0.9 V) peaking at −0.8 V (46%), with an HCOOH production rate of 73 ppm h^–1^, while the FE for the HER process is relatively small. Nevertheless, the FE_HCOOH_ at −0.5 V is much lower (11%) in comparison with that of rGO/Bi@Bi_2_O_3_. From these results, we can conclude that the metallic Bi core plays a critical role in anticipating the potential for HCOOH formation, albeit the fact that Bi_2_O_3_ is only present in the shell of the NP may be the cause for the modest current density, whereas in GO/Bi_2_O_3_ the amount of the Bi oxide is comparatively larger (NPs are fully oxidized). This is in line with previous observations during CO_2_RR by Bi_2_O_3_ catalysts that upon reduction to metallic Bi could generate higher HCOOH current densities.^31^ As we previously reported in the case of HER catalyzed by oxidized carbon nanotubes/titanium oxide nanocomposites, the carbon–metal oxide turns out to be less electrocatalytically active in the as prepared nanocomposites. In fact, in the fresh materials, the metal oxide is still in an amorphous state and the CNS presents too many COOH functional groups, leading to a less efficient electronic communication.^51^ This is also observed here in the case of the as prepared GO/Bi@BiO_*x*_ nanocomposite as compared to the two thermally treated catalysts, which show in general lower FE_HCOOH_ at any potential. The criticality of the CNS-MO intimate contact is also demonstrated by measuring the performances of the electrocatalyst made by physically mixing GO with Bi_2_O_3_ (Figure S11), which gave similar current density to those of GO/Bi_2_O_3_ but much worse electrocatalytic activities and lower stability than GO/Bi_2_O_3_. This result demonstrated not only that GO is instrumental to simply enhance the total current density by an overall improved conductivity (capacitive current) but also it contributes to an improved electron mobility toward the active Bi oxide sites, thus boosting the electrocatalytic performance.

**Figure 7:**
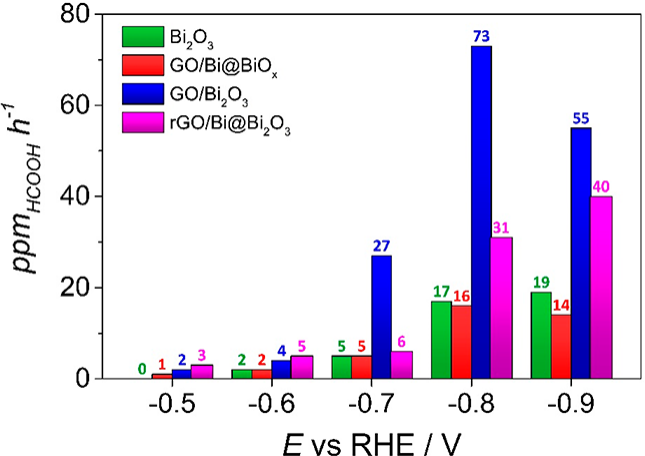
HCOOH productivity by the different catalysts. Formic acid production rate in ppm h^–1^ at different potentials for Bi_2_O_3_ (green), GO/Bi@BiO_*x*_ (red), rGO/Bi@Bi_2_O_3_ (pink), and GO/Bi_2_O_3_ (blue).

The formic acid production rate plot (Figure 7) notes the difference in the potential range for CO_2_RR selectivity: the HCOOH production is higher for rGO/Bi@Bi_2_O_3_ until −0.6 V, while GO/Bi_2_O_3_ becomes more active in the range −0.7 to −0.9 V. However, it is worth noting that, in terms of productivity, GO/Bi_2_O_3_ reaches a peak at −0.8 V and then there is a decline, whereas for rGO/Bi@Bi_2_O_3_ the HCOOH productivity increases linearly with moving toward more negative potential, despite the drop in selectivity. This is a result of the much higher current density achieved when using the rGO, and we presume that also the Bi metal core contributes to a better electron transfer toward the Bi_2_O_3_ active phase (see below). A comparison of HCOOH productivity with the free-standing bulk Bi_2_O_3_ indicates that the thermally treated nanocomposites are considerably more efficient at any explored potential, with maximum productivity by GO/Bi_2_O_3_ at −0.8 V being 4 times higher than bulk Bi_2_O_3_. Importantly, nanocomposites also displayed great chemical and mechanical stability with a constant FE% in prolonged electrosynthesis (>18 h, at −0.8 V; see Figure S12).

**Figure 8:**
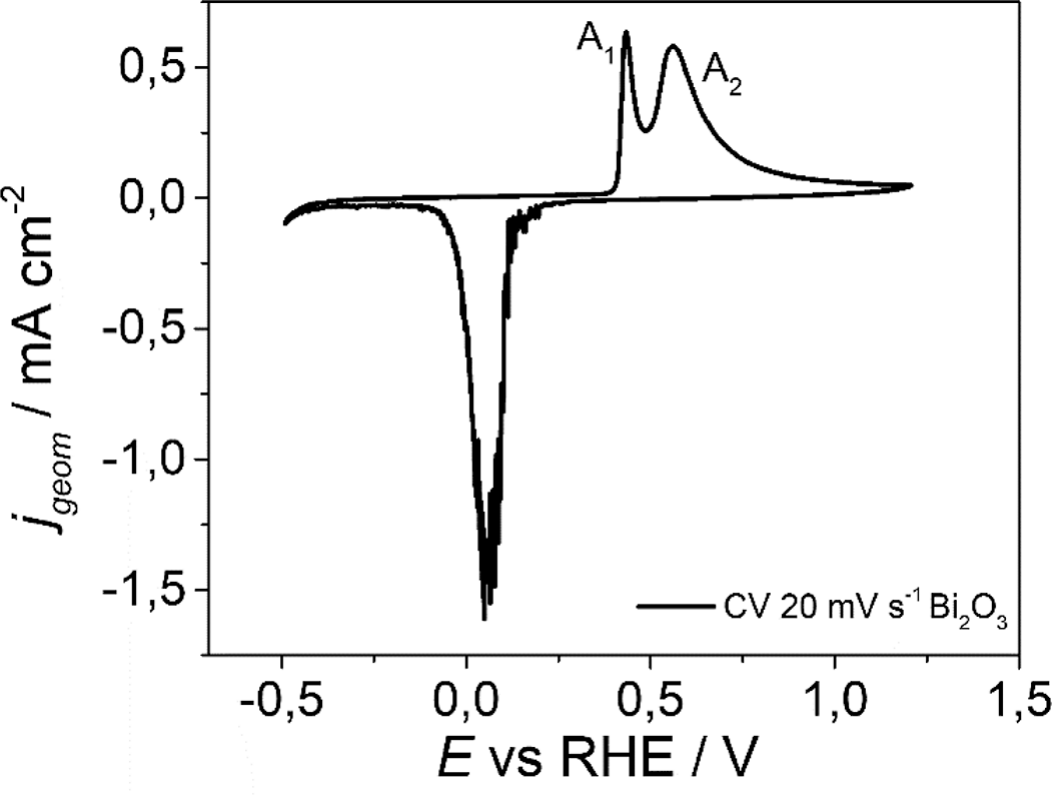
Cyclic voltammetry investigation. CV at 20 mV s^–1^ in Ar-saturated KOH 0.1 M of Bi_2_O_3_. Two peaks are distinct for the oxidation reaction: A_1_ is the oxidation of Bi metal sites presents in the porosity of nanoparticles, while the A_2_ peak is due to oxidation of Bi metal sites on nanoparticles surface. The potentials were corrected post measurement for the ohmic drop, considering the resistance of system.

The electrochemical peculiar features of Bi_2_O_3_ and the role of GO as well as of the metallic Bi in relation to CO_2_ activation were investigated by cyclic voltammetry (CV) using a three-electrode electrochemical cell.^52^ The electrochemical features of bulk Bi_2_O_3_ prepared by conventional synthesis was first studied. Figure 8 reports the CV of bulk Bi_2_O_3_ at 20 mV s^–1^ in Ar-saturated KOH 0.1 M.

Under an open circuit potential condition, the material is completely oxidized (*E*_oc_ = 0.89 V vs RHE). The bismuth oxide in contact with the alkaline solution undergoes a partial dissolution into the ionic species BiO_2_^–^.^52^ The cathodic peak is due in the main to reduction of dissolved species BiO_2_^–^, according to these reactions.^53^
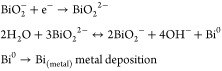


The oxidation peak A_2_ and the subsequent plateau are due to the oxidation of Bi metal to Bi(III). While the peak A_1_ is explained by Vivier et al. as the oxidation of small fraction of Bi metal sites to Bi(III) in the microporous cavities, where the amount of reactant is limited, the oxidation happens until exhaustion of OH^–^ species. In both cases, the electrochemical reactions follow these reaction mechanisms:^53^
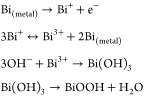


**Figure 9:**
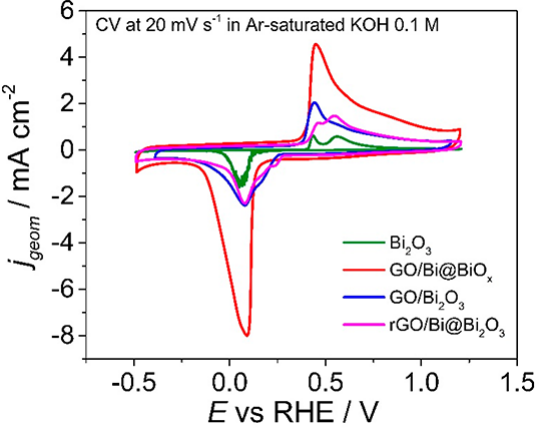
Cyclic voltammetry investigation. Cyclic voltammetries at 20 mV s^–1^ in Ar-saturated KOH 0.1 M of Bi_2_O_3_ (green), GO/Bi@BiO_*x*_ (red), GO/Bi_2_O_3_ (blue), and rGO/Bi@Bi_2_O_3_ (pink). The potentials were corrected post measurement for the ohmic drop, considering the resistance of the system.

When no potential is applied, the Bi(OH)_3_ and BiOOH evolve spontaneously to form Bi_2_O_3_. The CV of both composite GO/Bi_2_O_3_ and rGO/Bi@Bi_2_O_3_ were then compared with that of bulk Bi_2_O_3_ to gain insights into the role of the CNS as well as of the metallic Bi. As a first important observation, the Bi_2_O_3_ has a lower current than samples with carbon support, and as expected, the capacitance of the double layer is 100 times higher for both composites (Figure 9 and Figure S13). Interestingly, the Faradaic current for GO/Bi_2_O_3_ and rGO/Bi@Bi_2_O_3_ is approximately three times higher as compared with that of Bi_2_O_3_. Based on our previous endeavors on CO_2_RR catalyzed by CNS–metal oxide nanohybrids,^18,19^ we are confident in relating this effect to higher electrical conductivity of the GO, which reduces the internal system resistance and supplies electrons to the metal oxide, activating the Bi_2_O_3_ nanoparticles on support. In contrast, the resistance and aggregation of pure Bi_2_O_3_ can decrease the electrochemical active surface area.

The CNS support also increases the stability of the Bi_2_O_3_ nanoparticles. In fact, for a sample with pure Bi_2_O_3_, the faradaic current decreases with the increase in the number of cycles. It is possible to notice the different CV patterns between GO/Bi_2_O_3_ and rGO/Bi@Bi_2_O_3_, where in the first case the A_1_ peak is larger than A_2_, while for the sample with rGO, the A_1_ peak decreases in favor of the A_2_ peak. This observation well fits with the different structures of the two nanocomposites, whereby the metallic Bi core in the rGO/Bi@Bi_2_O_3_, and this particular core–shell motif plays a role in determining the electrochemical behavior. To this end, it is worth reporting previous findings with other core–shell electrocatalysts, such as Cu@SnO_2_, which exhibited variable selectivity depending on the thickness of the oxide layer, with theoretical calculations indicating that possible alloying of the SnO_2_ with Cu and resulting synergy, causing a compression of the oxide shell to trigger CO or HCOOH depending on the shell thickness.^54^ We finally performed a compositional optimization study of the GO/Bi_2_O_3_ to further boost HCOOH production rates. For this purpose, two additional samples were investigated, with a higher (GO/Bi_2_O_3_ 3:1) or lower Bi_2_O_3_ loadings (GO/Bi_2_O_3_ 5:1), respectively. Interestingly, although the current densities do not change among the three catalysts, the FE is not directly proportional to the amount of Bi_2_O_3_ present in the sample. In fact, the GO/Bi_2_O_3_ 4:1 catalyst shows the best performance for CO_2_RR, with FE_HCOOH_ as high as 46% at −0.8 V, where the normalized formic acid production rate is 484 ppm_HCOOH_ h^–1^ g_Bi2O_3__^-1^. Instead, with GO@Bi_2_O_3_ 3:1, bearing a higher amount of Bi_2_O_3_, FE_HCOOH_ never exceed 10% until −0.8 V, with an increment at −0.9 V (Figure S14). It can therefore be deduced that nonoptimized GO/Bi_2_O_3_ may favor the reduction of CO_2_ to formic acid upon a fine balancing between the number of active sites and the material resistance (higher, e.g., in GO@Bi_2_O_3_ 3:1 because of the relative lower amount of conductive GO). In line with it, GO@Bi_2_O_3_ 5:1 shows significantly poorer CO_2_RR performances, where the top FE_HCOOH_ is a mere 8% (at −0.9 V) and a greater selectivity for the HER process is instead observed, evidently deriving from the overriding HER activity of the GO, therein present in much larger relative amounts (Figure S14). It must be noted that, in all catalytic experiments, the intrinsic FE(HCOOH) of the Bi oxide active phase may be underestimated as at potentials more negative than −0.7 V, and there is also a contribution to HER by the graphene support, which cannot be avoided, as evaluated by screening the HER activity in CO_2_ by rGO at various potentials (Figure S15). Table S1 presents a comparison with other Bi-base electrocatalysts derived however from more complex synthetic procedures and under different electrochemical cell conditions.

## Conclusions

In conclusion, we carried out an in depth analysis on the role of GO when integrated with Bi_2_O_3_ nanoparticles in the electrocatalytic reduction of CO_2_, revealing remarkable differences depending on the structures. The presence of GO in intimate contact with the active phase (Bi_2_O_3_) causes a remarkable increase in the CO_2_RR performance toward the synthesis of formic acid. The results herein reinforce the concept that interfaces of MO with CNS promotes the activity and selectivity of the MO catalysts, offering an avenue to increase the electron trafficking nearby the MO, and contributing in a significant fashion to the long-term stability of the nanostructured catalysts. Differences were noted depending on the degree of oxidation of the carbon scaffold, with the rGO being more effective in favoring electron mobility as compared with the GO, thus facilitating electron transfers. Moreover, we prove that the adjustment of the hierarchy of the nanocomposite, by maintaining the metallic Bi(0) native core, gives rise to the formation of a double interfacial effect by the rGO and Bi(0) on the active Bi_2_O_3_ shell, resulting in a dramatic change of the catalytic behavior, such as an anticipation in the onset potential for CO_2_RR with concomitant higher FE_HCOOH_ at near-thermodynamic potential. The study lays fundamental aspects in the development of CNS/MO based electrocatalysts, although the absolute performance is yet to be optimized by adjusting the synthetic strategies and by increasing the mass transport (e.g., use of gas diffusion electrodes). In particular, the first aim will have to be to decrease the Bi@Bi_2_O_3_ particle size in order to (i) enhance the electroactive mass activity and (ii) enhance the carbon–metal oxide interface contact area. Further integration, within the nanocomposite, of a third metal phase could lead to coupled CO_2_RR mechanism in order to attempt the synthesis of more challenging (C_2+_) carboxylic acids.

## Supporting Information Available

TEM analysis, CO_2_RR catalytic performance differences between GO vs GO/Bi_2_O_3_, effect of the GO–MO interface on CO_2_RR performance, stability, capacitance of double layer of Bi_2_O_3_ and rGO/Bi@Bi_2_O_3_, the effect of Bi_2_O_3_ loading on CO_2_RR and rGO CO_2_RR performances (PDF)
