# Supplementary material for: Driving up the Electrocatalytic Performance for Carbon Dioxide Conversion through Interface Tuning in Graphene Oxide–Bismuth Oxide Nanocomposites
Source: ACS Appl Energy Mater. 2022 Oct 20;5(11):13356–66. doi: 10.1021/acsaem.2c02013 (PMC9710520; doi:10.1021/acsaem.2c02013)
Supplement: Supplementary file 1 — ae2c02013_si_001.pdf [file ae2c02013_si_001.pdf]

Supporting Information

Driving up the electrocatalytic performance for carbon dioxide conversion through interface tuning in graphene oxide-bismuth oxide nanocomposites

Michele Melchionna, Miriam Moro, Simone Adorinni, Lucia Nasi, Sara Colussi, Lorenzo Poggini, Silvia Marchesan, Giovanni Valenti,* Francesco Paolucci, Maurizio Prato, and Paolo Fornasiero**

TEM analysis

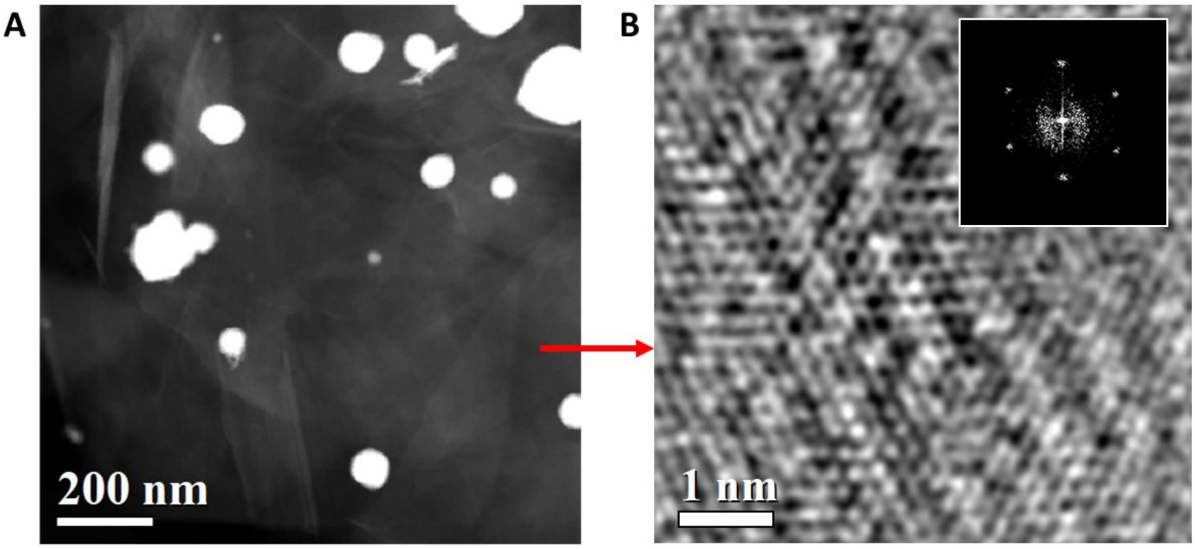

Figure S1. A) HAADF-STEM image of the Bi@BiO$_x$ NP dispersed on the GO, B) Filtered HRTEM image of a selected area in (red arrow) with the corresponding FFT in the inset, showing the typical 6-fold symmetry of a monolayer GO.

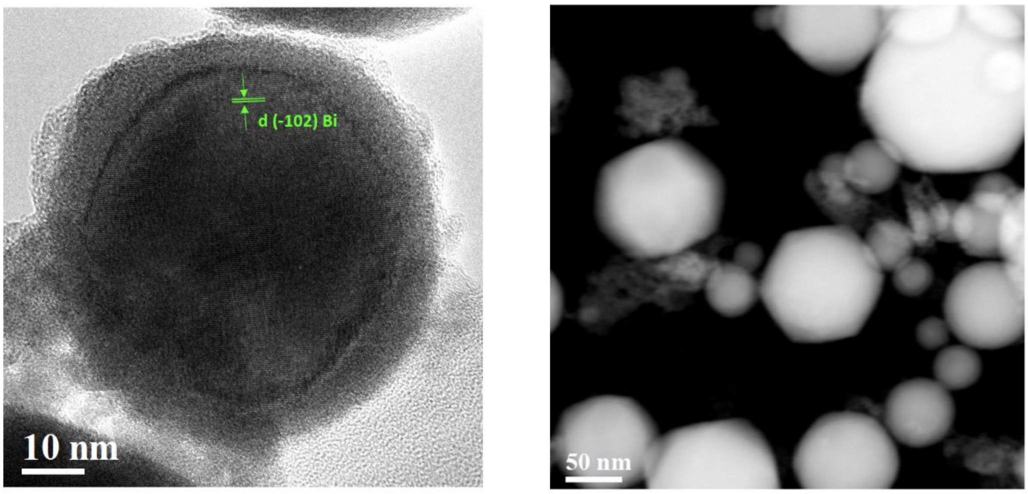

Figure S2. HRTEM of as prepared Bi@BiOx NP showing the core-shell configuration, with the d-spacing of the rombohendral Bi(0) core (left) and representative HAADF-STEM image (right)

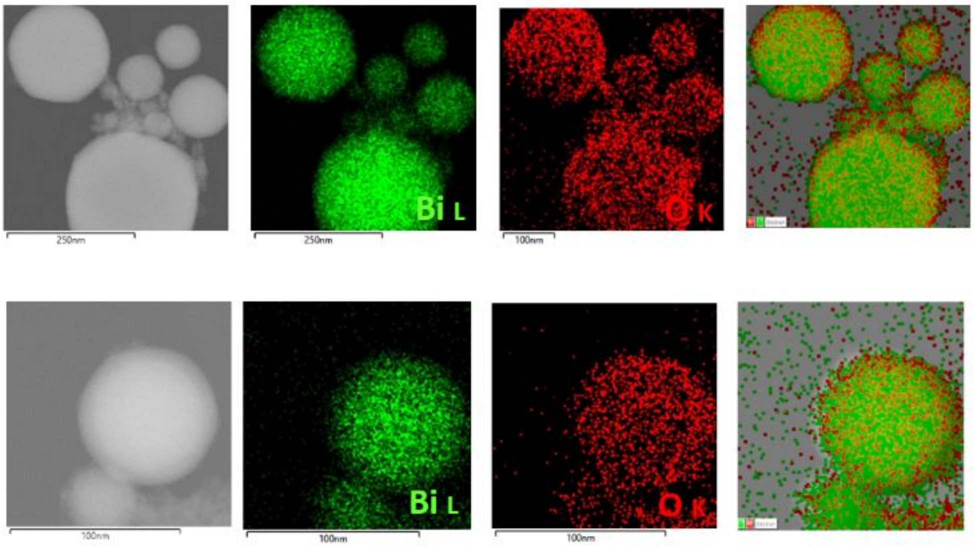

Figure S3. HAADF-STEM images of Bi@BiO_x NP with the corresponding Bi, O EDX maps (green and red) and the overlay showing that the BiOx is distributed around the shell.

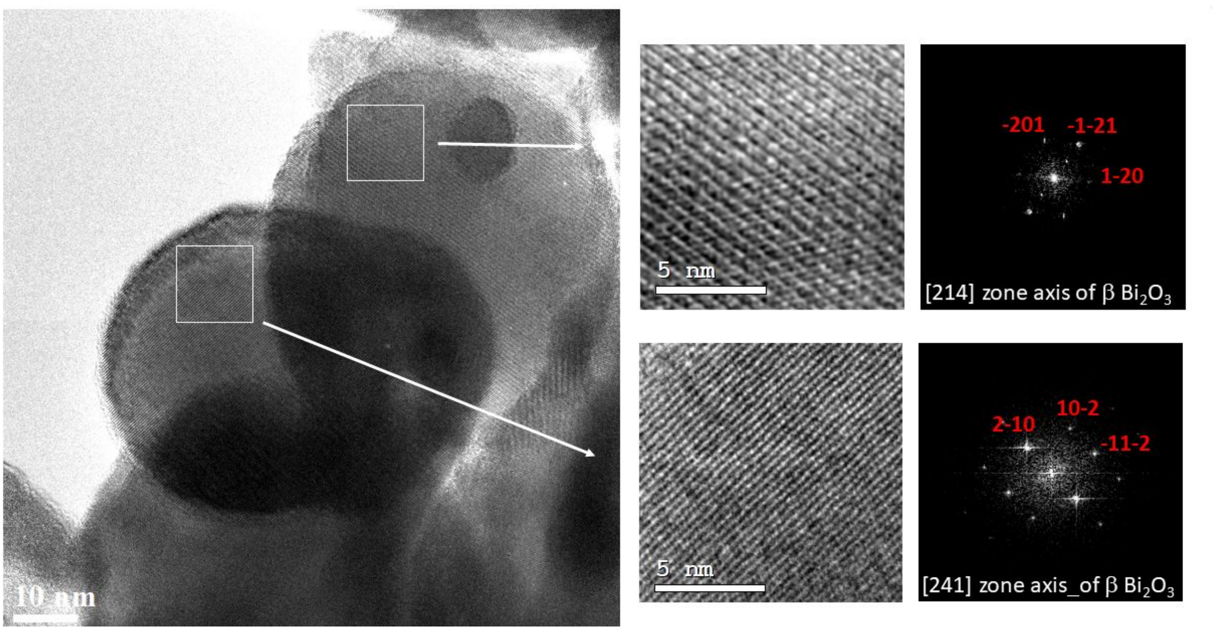

Figure S4. HRTEM of some Bi_2O_3 NP prepared by calcination of the Bi@BiOx (left). Enlarged HRTEM images of the two NPs with the corresponding FFTs showing the β-Bi_2O_3 phase in two different zone axes, confirming that the whole core has been oxidized

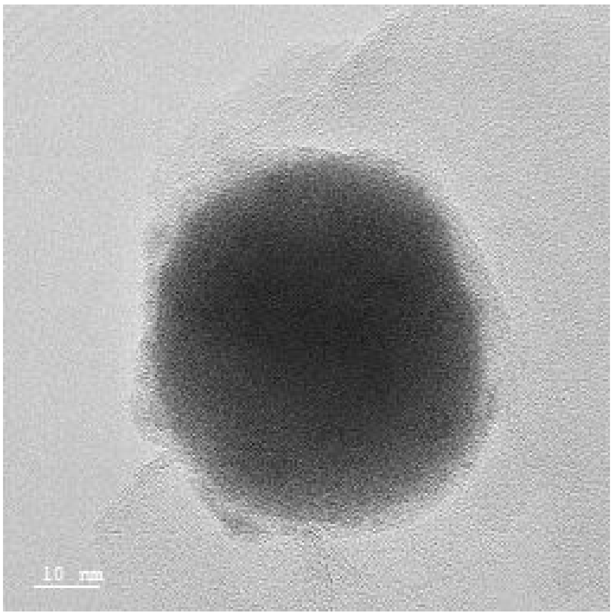

Figure S5. HRTEM of GO/Bi2O3 where it can be appreciated the coverage of the Bi2O3 NP with thin and small GO fragments.

some Bi_2O_3

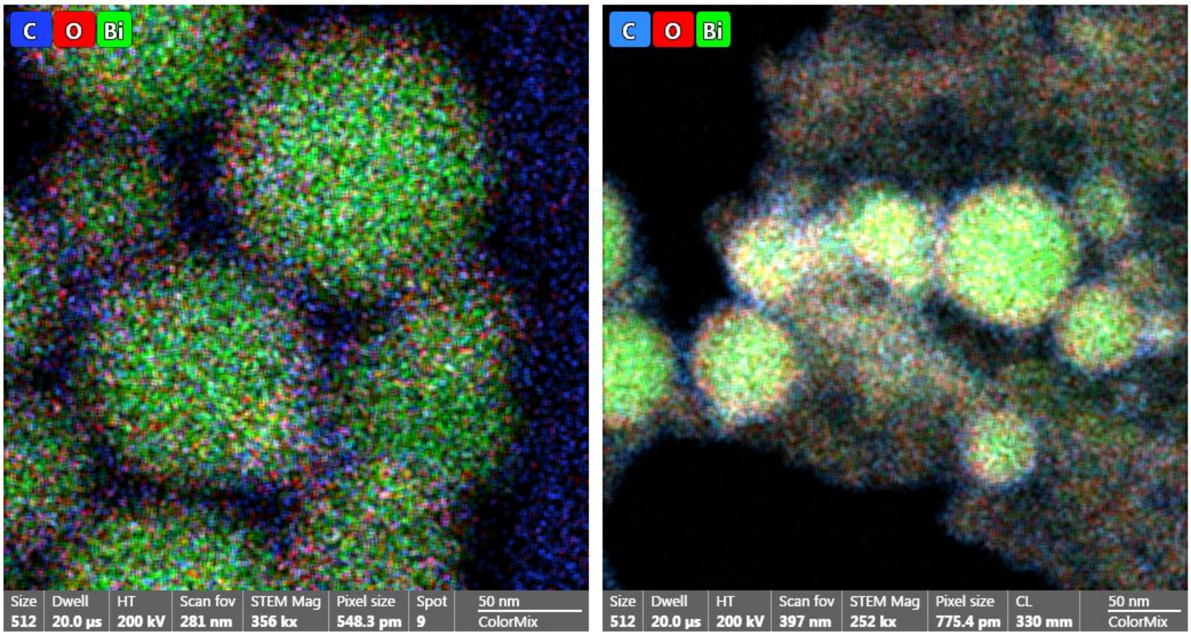

Figure S6. EDX mapping of the C, O and Bi atoms of GO/Bi_2O_3, showing that carbon (in the form of GO fragments) is spread arount the whole Bi_2O_3 NP.

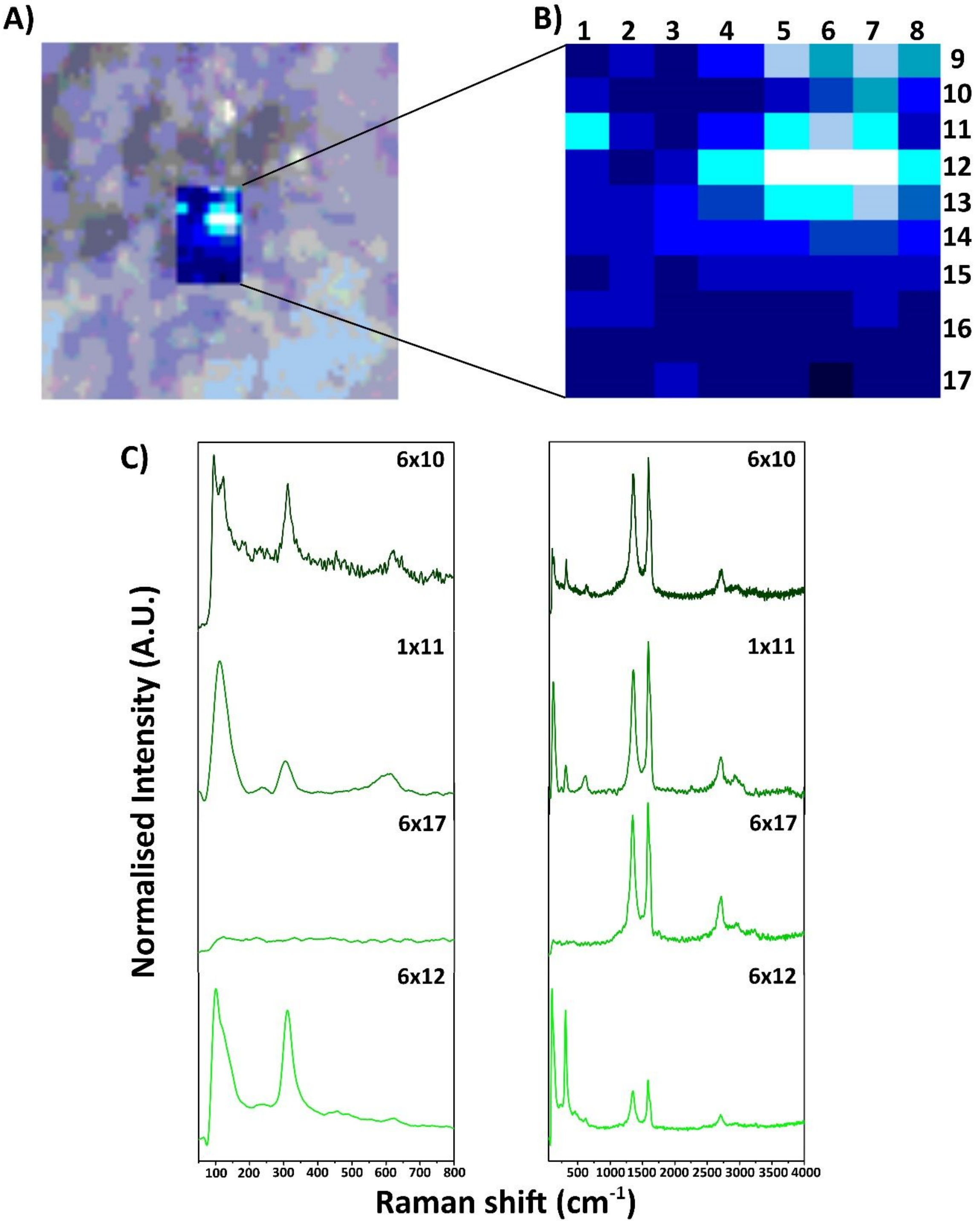

Figure S7. 80-point MicroRaman mapping of a selected area of the sample (top right: camera image; top left: expansion of the selected area) with corresponding Raman of 4 selected points of focus (bottom left: expansion in the Bi oxide Raman shift range; bottom right: full spectra)

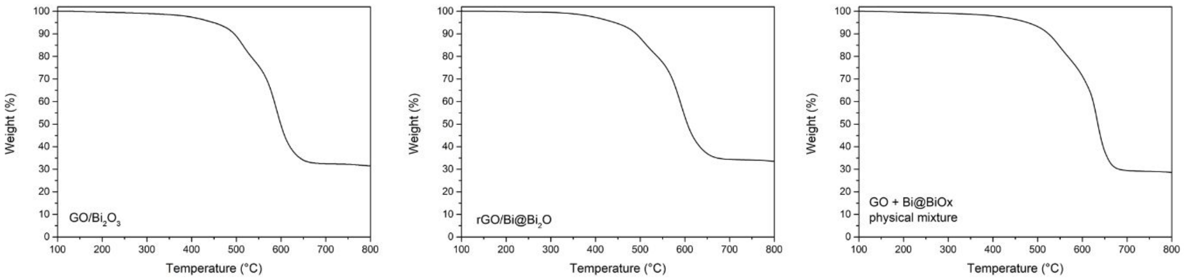

Figure S8. TGA of GO/Bi_2O_3, rGO@Bi@Bi_2O_3 and physical mixture of GO + Bi@BiO_x which confirms the closeness of the Bi oxide wt % with respect to nominal composition.

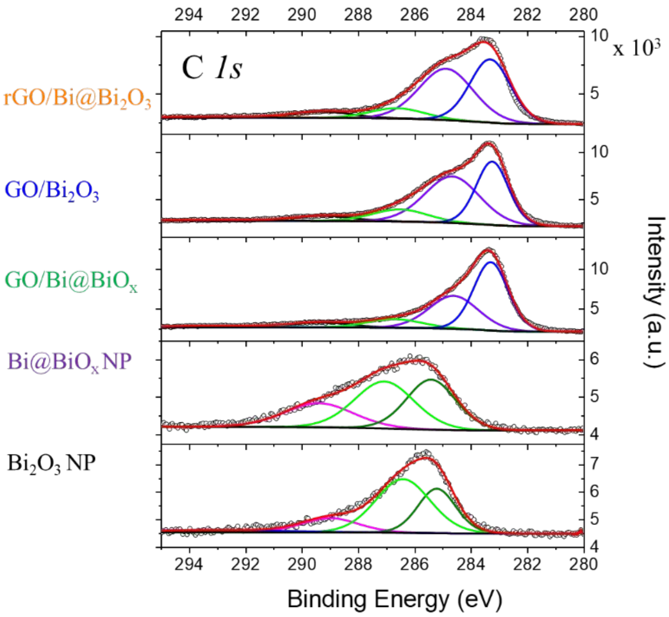

Figure S9. High-resolution XPS spectra in the C 1s region. Colours of the captions are associated to the materials as follows: black (Bi@BiO_x), green (GO/Bi@BiO_x), blue (GO/Bi_2O_3), orange (rGO/Bi@Bi_2O_3), purple (Bi_2O_3 NP).

CO_2RR catalytic performance differences between GO vs GO/Bi_2O_3

To be sure that the production formic acid is due to the catalytic activity of Bi_2O_3, the pristine GO was analysed in same condition of other samples. The FE for GO and GO/Bi_2O_3 are in figure S10.

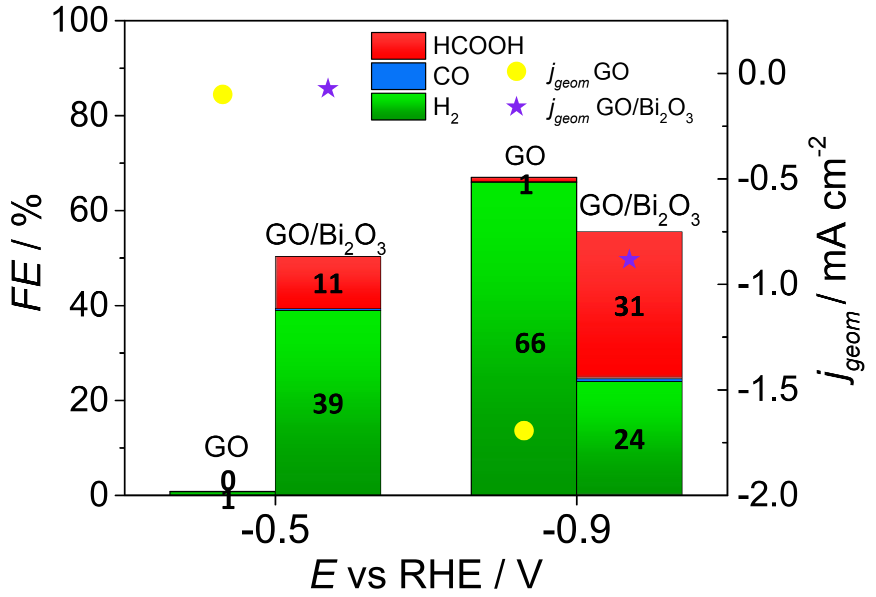

Figure S10. Faradaic Efficiency (FE) values are shown on left axis of detectable CO_2RR products: H_2 (green), CO (light blue) and HCOOH (red) for GO and GO/Bi_2O_3 at -0.5 V and -0.9 V vs RHE in CO_2-saturated $KHCO_3$ 0.5 M. The yellow

From the graphic is possible to notice how the GO is not able to produce the formic acid. The current density at -0.5 V vs RHE is the same for both samples, as evidence that the high current in the Bi-nanocomposites is mainly due to presence of GO, while the catalytic activity is attributed to bismuth oxide. This is evidence that GO is not active for CO_2RR, where the cathodic potential leads to the sole formation of hydrogen.

Effect of GO-MO interface on CO_2RR performance: GO/Bi_2O_3 (nanocomposite) vs GO + Bi_2O_3 (physical mixture)

Figure S11 reports the comparison between GO/Bi_2O_3, where the Bi_2O_3 nanoparticles were grown directly on GO, and physical mixture of GO and BiO_x, where the components were synthesized separately.

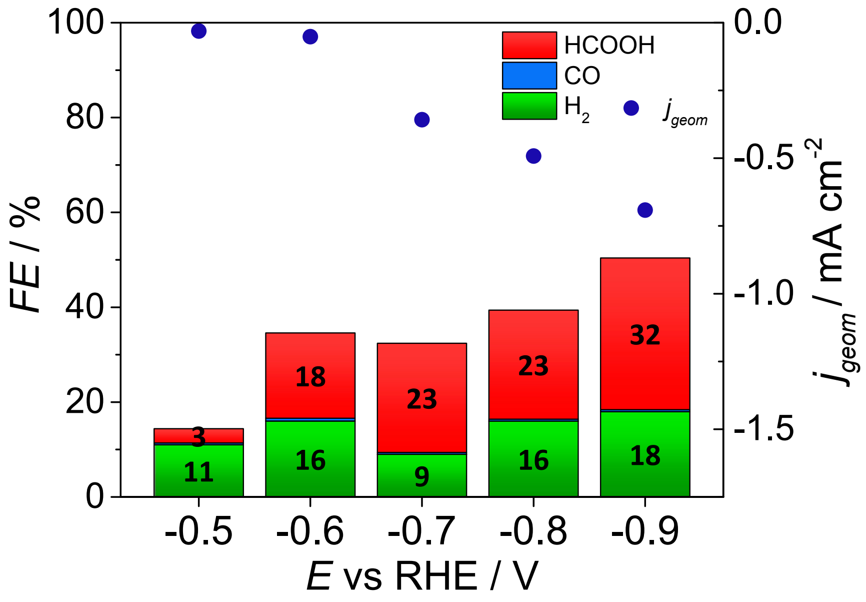

Figure S11. Faradaic Efficiency (FE) values are shown on left axis of detectable CO_2RR products: H_2 (green), CO (light blue) and HCOOH (red) for GO+BiO_x, in CO_2-saturated $KHCO_3$ 0.5 M. The blue points indicate the mean current density of sample

The current density the physical mixture sample is slightly lower than GO/Bi_2O_3, and also the formic acid production is lower. This is a proof that the good catalytic activity for CO_2RR derived by combination and interaction between GO and Bi_2O_3, and it is possible to obtain a good interaction only when the Bi_2O_3 is grown directly on GO.

Stability

The stability in the time of best sample was verified. The Chronoamperometry (CA) at -0.8 V vs RHE was applied for 2, 6, 12 and 18 hours at GO/Bi_2O_3 4:1 sample. The values of FE are in the figure S12.

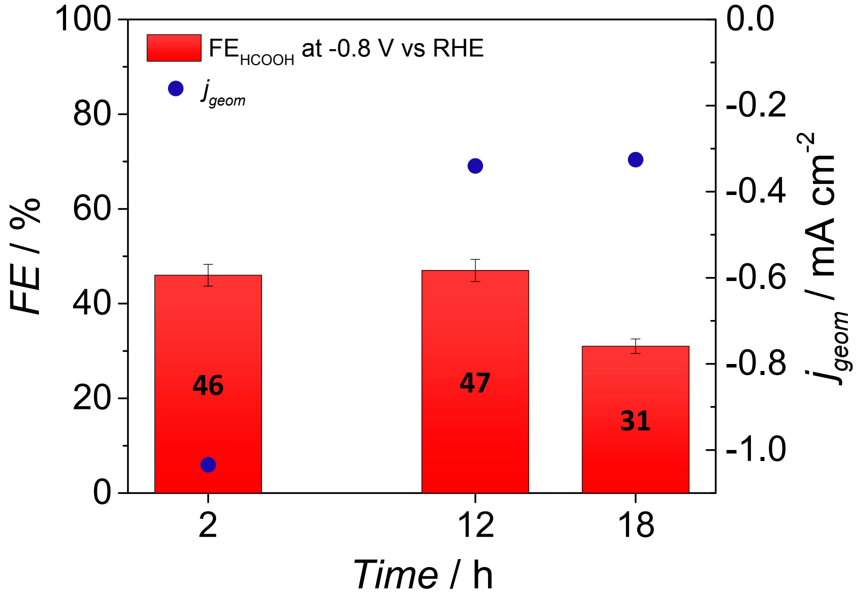

Figure S12. HCOOH Faradaic Efficiency and mean current density of GO@/i_2O_3 4:1 at -0.8 V vs RHE in CO_2-saturated $KHCO_3$ 0.5 M at different time (2,6, 12 and 18 h). The blue points indicate the mean current density of sample at that potential; the values are shown on right axis.

The FE_{HCOOH} does not change until at 12 h, then at 18 h there is decrease, (from 47 % to 31 %). This change in terms of FE might be associate to a partial aggregation or dissolution of Bi_2O_3 nanoparticles.

Capacitance of double layer of Bi_2O_3 and rGO/Bi@Bi_2O_3

The figure S13 shows the CVs of double layer for the samples Bi_2O_3 and RGO/Bi_2O_3. From the current density values as a function of the scan rate, it was possible to extract the double layer capacitance (C_{DL}) of two sample. With add of rGO on the sample, the capacitance increases of 100 times.

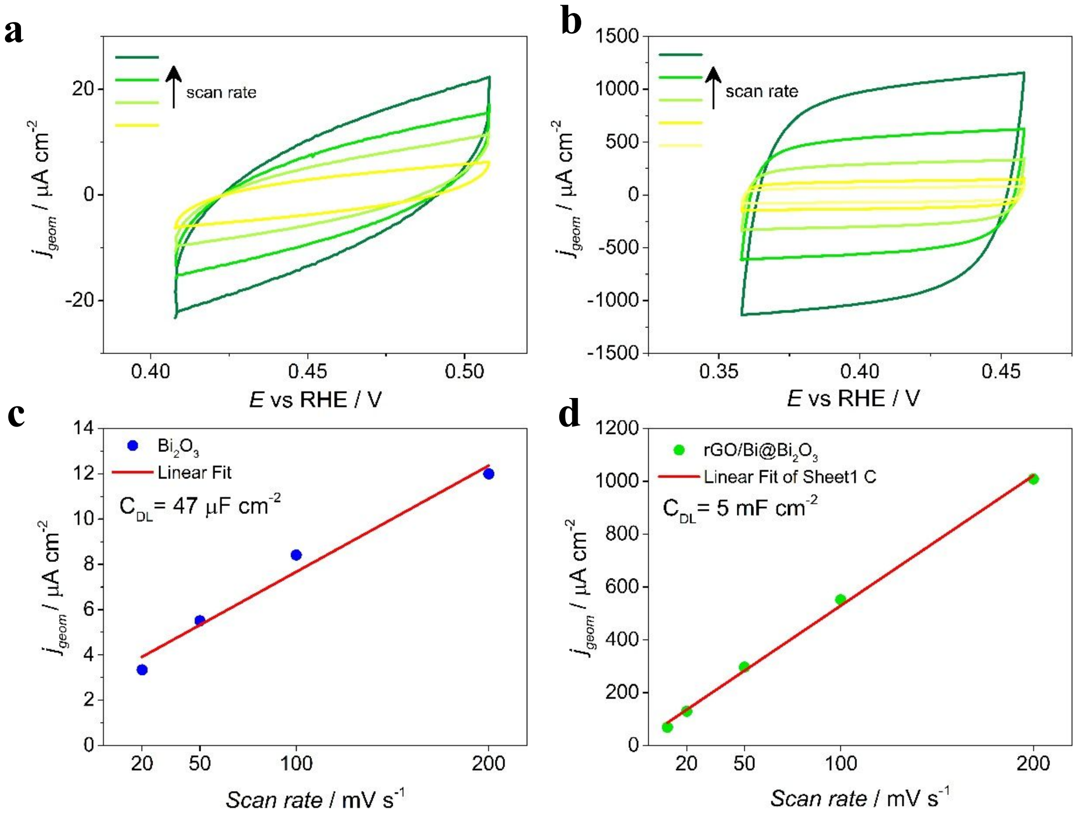

Figure S13. Measured CVs for **a** Bi_2O_3, **b** rGO/BI@Bi_2O_3 in Ar-saturated KOH 0.1 M. The current densities vs scan rate were plotted to extract the value of double layer capacitance (C_{DL}) of **c** Bi_2O_3 and **d** rGO/Bi@BI_2O_3. The current densities were obtained from the double layer charge/discharge curves at -0.45 V for Bi_2O_3 and -0.40 V for rGO/Bi@Bi_2O_3.

Effect of Bi_2O_3 loading on CO_2RR

The figure S14 shows the FE_{HCOOH} and the average current density at -0.5 V vs RHE and -0.9 V vs RHE for the samples with different loading of Bi_2O_3: GO/Bi_2O_3 3:1, GO/Bi_2O_3 4:1 and GO/Bi_2O_3 5:1.

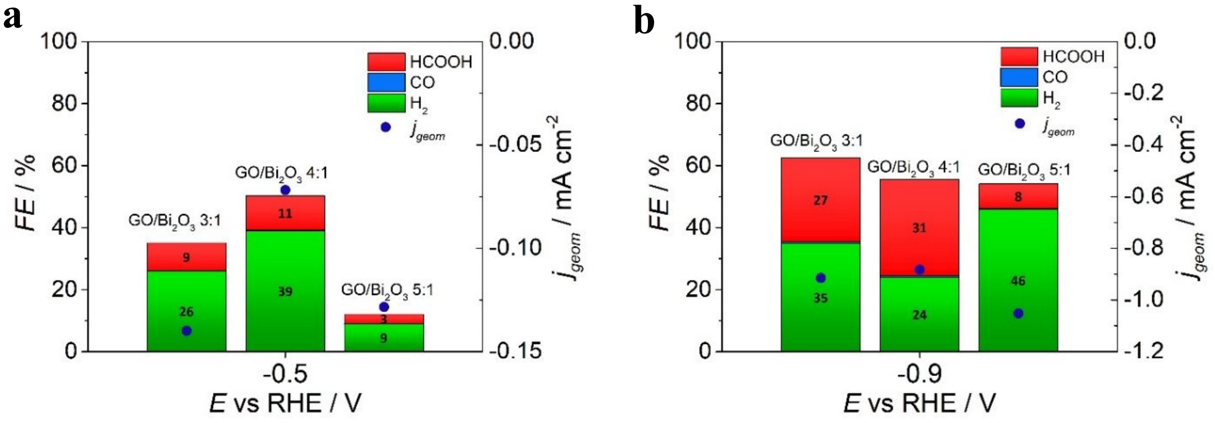

Figure S14 Faradaic Efficiency (FE) values are shown on left axis of detectable CO_2RR products: H_2 (green), CO (light blue) and HCOOH (red) for GO/Bi_2O_3 3:1, GO/Bi_2O_3 4:1 and GO/Bi_2O_3 5:1 a) at -0.5 V vs RHE and b) -0.9 V vs RHE in CO_2-saturated $KHCO_3$ 0.5 M. The blue points indicate the mean current density of sample at that potential; the values are shown on right axis.

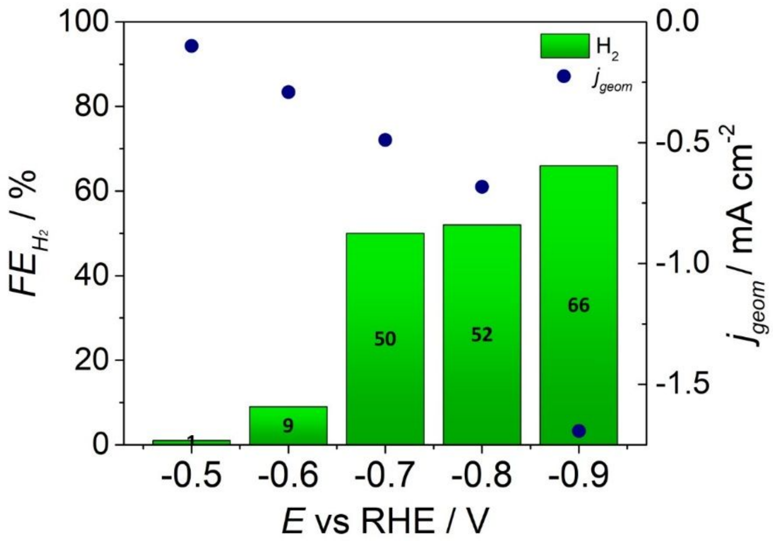

Figure S15. Faraday Efficiencies measured using only rGO as the electrocatalysts at different applied potentials. The blue points indicate the mean current density of sample at that potential; the values are shown on right axis.

Table S1. Comparison between the electrochemical performances of the nanohybrids with other recently reported Bi based materials active for the CO_2RR conversion towards HCOOC.

Catalyst	Potential (V vs RHE)	FE (%)	Reference
**rGO/Bi@Bi_2O_3**	**-0.5**	**38**	**This work**
**GO/Bi_2O_3**	**-0.8**	**46**	**This work**
**Bi NP@MWCNT**	-0.84	95	ACS Sustainable Chem. Eng. 2020, 8, 4871−4876
**Bi@Bi2O3 core–shell NPs in chloroplast porous carbon**	-1.0	94	Sci. Bull., 2020, 65(19), 1635–1642
**Bi2O3 NTs**	-0.74	89	Nat. Commun., 2019, 10, 2807
**Cu foam@BiNW**	-0.69	93	Energy Environ. Sci., 2019, 12, 1334–1340.
**Bi_2O_3@ carbon nanorods**	-1.1	93	Angew. Chem., Int. Ed., 2020, 59, 10807–10813
**Bi nanoparticles–PVP/ CC600**	-1.0	81	Applied Catalysis B: Environmental 284, 2021, 119723
**Bi-based MOF material**	-0.97	80	Adv. Funct. Mater.2020, 30, 1910408
**Bi/Bi2O3/NrGO-700 hybrid**	-0.9	85	Chinese Chemical Letters 31 (2020) 1415–1421
**Bi2O3 Nanosheets**	-1.4	94	Angew.Chem.Int.Ed.2019,58,13828–13833
**Dendrite Bi foil**	-0.74	89	ACS Catal. 2017, 7, 5071−5077
**Bi_2O_3 nanoparticles**	-1.2	91	ChemElectroChem2018, 5, 3741–3747

References

1. Verlato, E. *et al.* CO_2 reduction to formic acid at low overpotential on BDD electrodes modified with nanostructured CeO_2. *J. Mater. Chem. A* **7**, 17896–17905 (2019).

2. Hori, Y. *et al.* 'deactivation of copper electrode' in electrochemical reduction of CO_2. *Electrochim. Acta* **50**, 5354–5369 (2005).

3. Kas, R. *et al.* Electrochemical CO_2 reduction on Cu_2O-derived copper nanoparticles: Controlling the catalytic selectivity of hydrocarbons. *Phys. Chem. Chem. Phys.* **16**, 12194–12201 (2014).
